# Adipocytokines and Metabolic Syndrome in Patients with Schizophrenia

**DOI:** 10.3390/metabo10100410

**Published:** 2020-10-14

**Authors:** Irina A. Mednova, Anastasiia S. Boiko, Elena G. Kornetova, Daria A. Parshukova, Arkadiy V. Semke, Nikolay A. Bokhan, Anton J. M. Loonen, Svetlana A. Ivanova

**Affiliations:** 1 Mental Health Research Institute, Tomsk National Research Medical Center of the Russian Academy of Sciences, Aleutskaya str., 4, 634014 Tomsk, Russia; anastasya-iv@yandex.ru (A.S.B.); kornetova@sibmail.com (E.G.K.); susl2008@yandex.ru (D.A.P.); semkeniipz@tnimc.ru (A.V.S.); bna909@gmail.com (N.A.B.); ivanovaniipz@gmail.com (S.A.I.); 2 University Hospital, Siberian State Medical University, Moskovsky trakt, 2, 634050 Tomsk, Russia; 3 Department of Psychiatry, Addictology and Psychotherapy, Siberian State Medical University, Moskovsky trakt, 2, 634050 Tomsk, Russia; 4 Unit of PharmacoTherapy, Epidemiology, and Economics, Groningen Research Institute of Pharmacy, University of Groningen, 9713AV Groningen, The Netherlands; a.j.m.loonen@rug.nl

**Keywords:** schizophrenia, metabolic syndrome, adipocytokines, leptin, adiponectin, TNF-α, IL-6

## Abstract

The adipokines leptin, adiponectin, tumor necrosis factor-alpha (TNF-α), and interleukin 6 (IL-6) might be associated with metabolic syndrome (MetS) in patients with schizophrenia. In the present study, we attempted to confirm the results of previous reports and assessed their MetS-related correlation with body fat composition and biochemical parameters. We measured in 46 patients with schizophrenia and MetS serum levels of adiponectin insulin, leptin, TNF-α and IL-6 and compared these levels to those of patients with schizophrenia without MetS. The MetS patients had significantly increased leptin levels and leptin/adiponectin ratios, as well as decreased adiponectin levels. Leptin levels correlated with several metabolic parameters, both in patients with and without MetS, including body fat percentage, total fat fold, and body mass index (BMI). Patients without abnormal MetS components had lower levels of leptin and leptin/adiponectin ratios compared with patients who had one or two MetS components. Leptin/adiponectin ratios were higher in patients who had four rather than three MetS components. Multiple regression analysis revealed multiple associations for leptin but only one for adiponectin, TNF-α, and IL-6. Our results support an important pathophysiological role for leptin more than adiponectin in patients with schizophrenia with MetS.

## 1. Introduction

The prevalence of metabolic syndrome (MetS) amog patients with schizophrenia is high in comparison to the general population. In a meta-analysis of 77 clinical studies, including more than 25,000 patients with schizophrenia, Mitchell et al. [1] reported a prevalence of 32.5%. MetS is associated with an increased risk for type 2 diabetes and cardiovascular pathology and, therefore, with high mortality rates among patients with schizophrenia [2,3,4]. In a recent study, our group showed an increase in body fat composition after six weeks of therapy using second-generation antipsychotics (SGAs) with a magnitude that depended upon the presence of MetS [5]. The endocrine functions of adipose tissue are widely known for their ability to produce bioactive peptides and cytokines, or so-called “adipokines,” which regulate metabolism and inflammation and may decrease the insulin sensitivity of tissues [6,7,8,9]. Chronic, low-grade inflammation during obesity has been established to represent a risk factor for psychiatric disorders [10,11].

Leptin was discovered in 1994 and is one of the most studied adipokines [12]. Leptin’s main function is to suppress appetite after reaching satiety and to stimulate the body to expend energy by activation of leptin receptors in the central nervous system, in particular, those localized within the hypothalamus as well as within the peripheral receptors located in adipose tissue, skeletal muscle, the pancreas, and other tissues and organs [13,14,15]. Apart from a range of metabolic effects [14,15], leptin is linked by many other pathophysiological effects to cardiovascular morbidity [16,17]. Remarkably, elevated leptin levels have also been observed in patients with schizophrenia [18,19].

Adiponectin is an adipocyte-derived plasma protein that was discovered in 1995 [12]. It plays a beneficial role in lipid and glucose metabolism, has anti-diabetic, anti-atherosclerotic, and anti-inflammatory properties, and is considered to be a potential biomarker of MetS [20,21]. A meta-analysis showed that adiponectin can serve as a moderately accurate predictor of MetS [22]. Especially, a high leptin–adiponectin ratio appears to be a suitable biomarker for metabolic syndrome [23,24,25].

In addition to leptin and other adipokines, pro-inflammatory cytokines are generated in adipose tissue as well [6,16,26]. In 1993, a link was found between increased levels of pro-inflammatory cytokines and tumor necrosis factor-alpha (TNF-α) produced by adipose tissue with insulin resistance [27].

TNF-α plays an important role in inducing obesity-related insulin resistance, which is an important mechanism of metabolic syndrome [28,29,30]. Interleukin 6 (IL-6) is a second pro-inflammatory cytokine produced by adipocytes and plays a role in mediating insulin resistance [31,32]. Both IL-6 and TNF-α are capable of inhibiting the synthesis of adiponectin from adipose tissue [33,34]. These pro-inflammatory adipokines (i.e., TNF-α and IL-6) have been found to be associated with MetS [35]. In addition, a meta-analysis showed positive correlations for leptin and negative correlations for adiponectin with TNF-α and IL-6 [36]. Moreover, schizophrenia is known to be associated with abnormal inflammatory activation [11,37,38].

In the present study, we aimed to confirm the results of previous reports of increased serum cytokines and adipokine levels in patients with schizophrenia [39], as well as their association with metabolic parameters [18,40]. In addition, we wanted to assess the possible correlation of adipokine levels with body fat composition and biochemical parameters depending on the presence of MetS.

## 2. Results

Included were 110 patients with schizophrenia (Table 1), 46 of whom (41.8%) had MetS while the other 64 (59.2%) did not. We found a significant difference between the two groups concerning age (*p* = 0.001), duration of illness (*p* = 0.001), and the majority of the biochemical and anthropometric parameters.

We found significant decreases in the serum concentrations of adiponectin (*p* = 0.002) and increases in the leptin serum levels (*p* < 0.001) and the leptin–adiponectin ratios (*p* = 0.002) in patients with schizophrenia with MetS in comparison to patients without MetS (Table 2).

As the sample of patients without MetS contained both metabolically completely healthy subjects and subjects with abnormal metabolic parameters, and the patients with MetS included both subjects with several abnormal metabolic parameters and subjects with completely altered metabolic components, we evaluated the association between adipocytokine levels and the number of MetS components in patients with schizophrenia. Statistically significant differences were found in leptin levels among patients with zero and two MetS components (*p* = 0.0146) and a leptin–adiponectin ratio among patients with zero and one MetS components (*p* = 0.0147) (Table 3).

Due to the small number of patients who registered with all five criteria for MetS (*n* = 5), their data were not taken into account in the statistical analysis. These patients had an adiponectin level of 6.86 (5.14; 16.42) μg/mL, a leptin level of 25.7 (22.6; 27.77) pg/mL, a leptin–adiponectin ratio of 3.29 (2.44; 5.00), a TNF-α level of 16.15 (14.27; 21.09) pg/mL, and an IL-6 level of 5.75 (4.2; 6.31) pg/mL.

In patients with MetS, we found statistically significant differences in the leptin–adiponectin ratios among patients with three and four MetS components (*p* = 0.0041) (Table 4).

**Figure 1:**
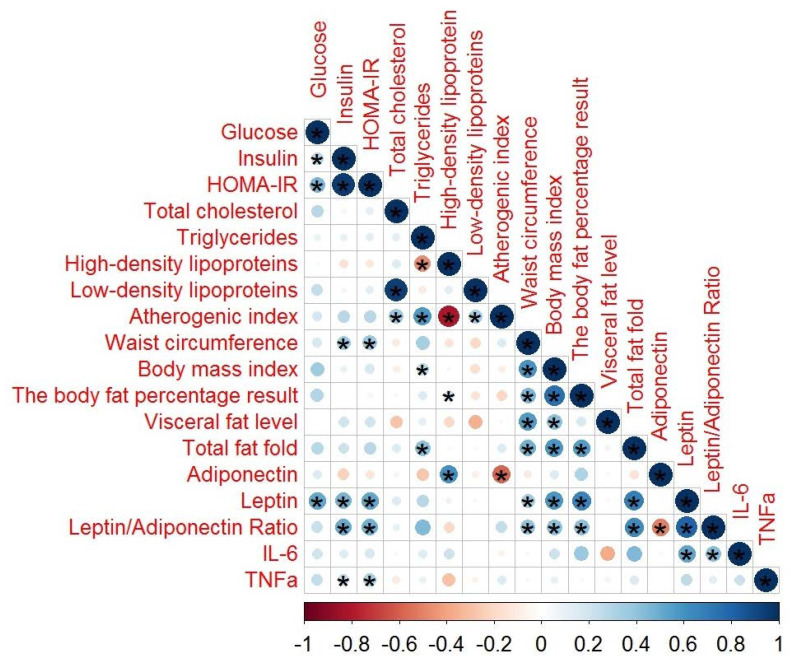
Spearman’s correlation analysis of the adipocytokines in patients with schizophrenia with MetS. In this figure, the red and black circles refer to negative and positive correlations, respectively; * *p* < 0.05. The size and color intensity of the circles are proportional to the correlation coefficient. In the legend at the bottom, the intensity of the color shows the rate of correlations and the corresponding relationships.

**Figure 2:**
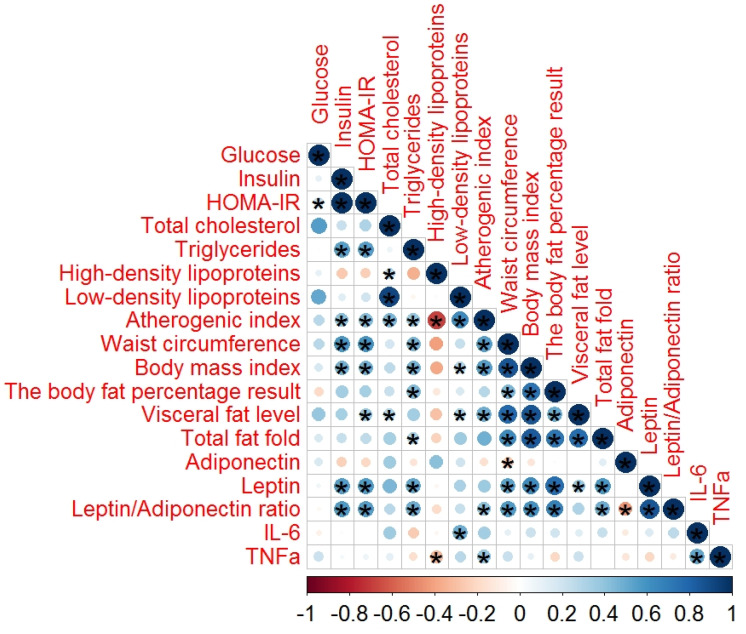
Spearman’s correlation analysis of the adipocytokines in patients with schizophrenia without MetS. In this figure, the red and black circles refer to negative and positive correlations, respectively; * *p* < 0.05. The size and color intensity of the circles are proportional to the correlation coefficient. In the legend at the bottom, the intensity of the color shows the rate of correlations and the corresponding relationships.

In order to evaluate the role of adipocytokines in schizophrenia, we also performed a correlation analysis of the serum adipocytokines, lipid profiles, glucose profiles, and anthropometric parameters in the 46 patients with schizophrenia with MetS (Figure 1) and the 64 without MetS (Figure 2).

In patients with MetS, a significant number of correlations with biochemical and anthropometric parameters of various strengths was found (Table A1). Additionally, we found that leptin levels correlated to a low degree but were still statistically significantly correlated with IL-6 (*r* = 0.382, *p* = 0.010). Leptin–adiponectin ratios showed a significantly low correlation with IL-6 (*r* = 0.420, *p* = 0.02). We did not find any correlations between IL-6 and the other parameters of metabolic status.

The correlations between the serum adipocytokines, lipid profiles, glucose profiles, and anthropometric parameters in patients with schizophrenia without MetS are illustrated in Figure 2.

In the group of patients without MetS, high-strength correlations were found between leptin and several anthropometric parameters, while low-strength correlations were found between the biochemical parameters, and adiponectin and the leptin–adiponectin ratios showed a significant moderate and a low correlation (Table A2). Additionally, IL-6 showed a moderate correlation with TNF-α *(r* = 0.539, *p* < 0.001).

Additionally, we performed a multiple regression analysis, considering leptin as a dependent variable and the statistically significant correlated metabolic parameters as independent variables. We found that after controlling for MetS, gender, and age, leptin was associated with glucose (β = 0.278, *p* = 0.002), waist circumference (β = 0.592, *p* = 0.0001), body mass index (BMI) (β = 0.570, *p* = 0.0001), body fat percentage (β = 0.603, *p* = 0.0001), visceral fat level (β = 0.344, *p* = 0.003), total fat fold (β = 0.532, *p* = 0.0001), total cholesterol (β = 0.171, *p* = 0.045), insulin (β = 0.352, *p* = 0.0001), and homeostasis model assessment of insulin resistance (HOMA-IR) (β = 0.334, *p* = 0.0001). Adiponectin, after adjusting for MetS, gender, and age, was associated only with waist circumference (β = −0.299, *p* = 0.035). The leptin–adiponectin ratio, after controlling for MetS, gender, and age, was associated with insulin (β = 0.389, *p* = 0.0001), HOMA-IR (β = 0.417, *p* = 0.0001), waist circumference (β = 0.579, *p* = 0.0001), BMI (β = 0.558, *p* = 0.0001), body fat percentage (β = 0.570, *p* = 0.001), total fat fold (β = 0.503, *p* = 0.0001), triglycerides (β = 0.300, *p* = 0.041), atherogenic index (β = 0.354, *p* = 0.008), and visceral fat level (β = 0.445, *p* = 0.004). After performing multiple regression analysis, IL-6 and TNF-α did not maintain an independent association with the other parameters (data not shown).

## 3. Discussion

In our study, we showed that leptin levels and leptin–adiponectin ratios were significantly increased and adiponectin levels significantly decreased in patients with schizophrenia with MetS in comparison to patients without MetS. However, we only found changes in leptin levels and leptin–adiponectin ratios in patients without MetS who had one or two components of MetS.

Correlation and multiple regression analyses showed that the involvement of leptin was predominantly in glucose metabolism. Adiponectin appears to have a more limited role. No important differences were observed concerning IL-6 and TNF-α levels.

As would be expected, the two groups of patients were different in many regards. The patients with MetS were significantly older and had a significantly longer illness duration, which is probably related to longer exposure to antipsychotic treatment. The observed difference, with respect to glucose and lipid metabolism as well as the fat content, was probably directly related to the presence of MetS itself.

Among adipocytokines, we found significant changes in the levels of leptin and adiponectin and their ratios in patients with schizophrenia with MetS. In patients without psychiatric diseases, the relationship between adiponectin and serum lipids appeared to be independent of body mass [41]. In our study, we found associations between adiponectin and lipid parameters only in patients with schizophrenia with MetS and only concerning high-density lipoprotein levels. Adiponectin is known to stimulate fatty acid oxidation—activation of lipoprotein lipase—thereby reducing plasma triglyceride levels [6], but this was not observed to significantly affect the levels in the present study.

According to the literature, leptin but not adiponectin is associated with the amount of visceral adipose tissue [42]. In our study, leptin levels were correlated with several metabolic parameters, both in patients with and without MetS, including body fat percentage, total fat fold, and BMI. However, only patients with schizophrenia but without MetS demonstrated a relationship between leptin and visceral fat levels. In other studies of patients with schizophrenia, both adiponectin and leptin levels were found to be associated with most metabolic parameters, which may be due to the fact that the assessment did not take into account the presence of MetS [18,40].

In our study, we did not find any important differences between TNF-α and IL-6 depending on the presence or number of components of MetS. An increase in the level of pro-inflammatory cytokines, including IL-6 and TNF-α, in patients with chronic schizophrenia is considered evidence of the key role of the activation of the immune system in the pathogenesis of the disease [43]. From this perspective, the absence of differences in the levels of IL-6 and TNF-α among the studied groups can be explained by the crossover effects of the presence of schizophrenia and metabolic syndrome. This is supported by studies that have demonstrated that increased levels of IL-6 and TNF-α are associated with the disease state itself and not the MetS components [39]. However, correlation analysis showed significant associations of these cytokines with aspects of lipid metabolism in patients without MetS and with parts of glucose metabolism in patients with MetS. TNF-α is known to contribute to an imbalance in cholesterol metabolism by cholesterol absorption and decreasing cholesterol efflux [44]. The role of TNF-α and IL-6 in insulin resistance has remained controversial. TNF-α infusion impairs glucose uptake in human skeletal muscle [45], but the subsequent injection of a human anti-TNF-α antibody has no effect on insulin sensitivity in obese type 2 diabetic patients [46]. IL-6 knockout mice have been shown to develop impaired glucose tolerance and obesity, which can be partially reversed by the administration of IL-6 [47]; on the other hand, even a 100-fold increase in IL-6 levels during post-absorptive conditions does not appear to affect the rate of change in glucose levels in humans [46].

In a previous paper, we observed that apolipoprotein A2 was lower in patients with schizophrenia without MetS, unlike patients with MetS [48]. Apolipoprotein A2 is the second most abundant protein in high-density lipoproteins and has potent anti-inflammatory effects [49]. Serum cortisol levels, another endogenous anti-inflammatory substance, have been shown not to significantly differ between patients with schizophrenia with or without MetS [50]. Although TNF-α correlates with high-density lipoprotein levels, our findings indicate a lack of influence of the pro-inflammatory IL-6 and TNF-α upon the readiness to develop MetS. In addition, we did not find a relationship between body fat components and cytokine levels in spite of the existence of evidence that IL-6 and TNF-α are overexpressed in adipose tissue [51].

The limitation of our study was that patients in both groups had suffered from schizophrenia for a long duration; therefore, we could not exclude the effect of antipsychotic therapy on the studied metabolic parameters. In addition, the data on the relationship between MS components and adipocytokines should be interpreted carefully due to the small sample of patients.

In conclusion, our results indicate that adiponectin and leptin may play a role in the pathophysiology of metabolic syndrome in patients with schizophrenia. However, the impact of adiponectin appears to be minor to that of leptin. We found that the leptin–adiponectin ratio was associated with a greater number of metabolic abnormalities in patients with schizophrenia compared to leptin or adiponectin alone, suggesting from the data in [18] that this ratio is the preferential biomarker of MetS in patients with schizophrenia. We did not find good evidence for the roles of pro-inflammatory cytokines IL-6 and TNF-α. The dysregulation of the metabolism, leading to metabolic syndrome, may be independent of the alterations within the immune system, including the cytokine network, which is hypothesized to be associated with the pathophysiology of schizophrenia [11,37,38].

## 4. Materials and Methods

### 4.1. Participants

The study was designed and implemented according to ethical principles for medical research involving human subjects, and was approved by the Local Bioethics Committee of the Mental Health Research Institute, Tomsk, Russia, (N187, 24 April 2018). Patients between the ages 18 and 55 were recruited from the Inpatient Department of Mental Health Research Institute and clinics from the Siberian State Medical University (Tomsk, Russia) after providing written informed consent. One hundred and ten patients with a clinical diagnosis of schizophrenia established in accordance with the International Statistical Classification of Diseases and Related Health Problems, 10th Revision (ICD-10: F20), were included in the study. Most patients received antipsychotic therapy in anti-relapse and maintenance dosages. Non-adherence to prescribed medication preceded readmission of patients and inclusion in research.

The exclusion criteria were the presence of comorbid neurological and somatic diseases that complicated an objective assessment of the clinical condition caused by the underlying disease: the presence of acute and chronic infectious, inflammatory, and autoimmune diseases; a lack of desire or ability to comply with the requirements of the research protocol; the use of psychoactive substances; taking medications that can affect metabolic parameters, for example, lipid-lowering and hypoglycemic agents.

Forty-six patients met the criteria for MetS according to the International Diabetes Federation (IDF) [52]. All of them showed signs of central obesity (i.e., waist circumference more than 94 cm in men and more than 80 cm in women) and the presence of any two of the following four signs:

The concentration of triglycerides in the serum was higher than 1.7 mmol/L (150 mg/dL) or lipid-lowering therapy was carried out;

1. The concentration of high-density lipoprotein in the serum was below 1.03 mmol/L (40 mg/dL) in men and 1.29 mmol/L (50 mg/dL) in women;
2. The arterial blood pressure level was systolic above 130 mmHg or diastolic above 85 mmHg (or with treatment of previously diagnosed hypertension);
3. Serum glucose concentration was greater than 5.6 mmol/L (100 mg/dL) (or previously diagnosed with type 2 diabetes).

### 4.2. Anthropometric Examination

Anthropometric parameters included waist circumference, BMI, body fat percentage, visceral fat, and total fat fold. Waist circumference was measured with a measuring tape. The body fat percentage, visceral fat level, body weight, and body mass index were determined using non-invasive bio-impedance analysis with the body composition and body fat monitor Omron BF508 (Omron Healthcare, Kyoto, Japan). Indicators of subcutaneous fat (total fat fold) were determined by electronic caliper. The total fat fold consisted of the sum of the fat folds in the shoulder, back, abdomen, and lower leg.

### 4.3. Laboratory Examination

#### 4.3.1. Blood Sampling

Blood was drawn by antecubital venipuncture in the first days of hospitalization after 12 h overnight fasting into BD Vacutainer^®^ with a clot activator (CAT) (BD, Franklin Lakes, NJ, USA). Blood samples with the CAT were centrifuged at 2000× *g* at 4 °C for 30 min; then, the serum was extracted and stored at −80 °C until analysis.

#### 4.3.2. Biochemical Parameters

Serum concentrations of glucose, triglycerides, high-density lipoproteins, and total cholesterol were measured by colorimetric enzymatic methods using commercial kits (Cormay, Łomianki, Poland). The concentration of low-density lipoprotein in the serum was calculated using the Friedewald equation [53].

The atherogenic index was calculated based on the concentration of cholesterol and HDL [54]. The concentration of insulin was determined on the multiplex analyzer MAGPIX (Luminex, Austin, TX, USA) using xMAP^®^ Technology with the use of the multi-analyte panel HMHMAG-34K by MILLIPLEX^®^ MAP (Merck, Darmstadt, Germany). The detected information was processed by the special software Luminex xPONENT^®^ with the subsequent export of data to the MILLIPLEX^®^ Analyst 5.1 program. The insulin concentration was determined in picograms per milliliter, and conversion to microunits per milliliter was carried out to calculate HOMA-IR. HOMA-IR was calculated to estimate insulin resistance using the Matthews equation [55].

#### 4.3.3. Adipocytokines Concentration

The concentration of leptin was determined on the multiplex analyzer MAGPIX (Luminex, Austin, TX, USA) using xMAP^®^ Technology with the use of the multi-analyte panel HMHMAG-34K by MILLIPLEX^®^ MAP (Merck, Darmstadt, Germany). The concentration of adiponectin was determined with use of the enzyme-linked immunosorbent assay kit (Cloud-Clone Corp. (CCC, USA), Houston, TX, USA).

The concentrations of TNF-α and IL-6 were determined on the multiplex analyzer MAGPIX (Luminex, USA) using xMAP^®^ Technology with the multi-analyte panel HCYTMAG-60K-PX41 by MILLIPLEX^®^ MAP (Merck, Darmstadt, Germany). The detected information was processed by the special software Luminex xPONENT^®^ with the subsequent export of data to the MILLIPLEX^®^ Analyst 5.1 program.

The multiplex analyzer MAGPIX is based at The Core Facility “Medical genomics” Tomsk National Research Medical Center.

### 4.4. Statistics

Statistical analysis was performed using SPSS 20.0 software for Windows. The Shapiro–Wilk test was applied to discern whether the data followed a normal distribution. The significance of intergroup differences was evaluated using the non-parametric Mann–Whitney *U* test for independent samples. The chi-square test was used to analyze the categorical variables. Descriptive statistics are presented as the median with 25% and 75% quartiles, Me (Q1; Q3). Correlation analysis was carried out with the use of Spearman’s rank test, and correlation coefficients were interpreted with the method of Mukaka [56]. The size of the correlations were encoded as follows: 0.00–0.30 as “negligible correlation”; 0.30–0.50 as “low correlation”; 0.50–0.70 as “moderate correlation”; 0.70–0.90 as “high correlation”; and 0.90–1.00 as “very strong correlation”. Multiple regression analyses were performed considering leptin, adiponectin, the leptin–adiponectin ratio, TNF-α, and IL-6 as the dependent variables and the statistically significant correlated metabolic parameters as the independent variables, provided the histogram of the distribution of the residues coincided with a normal distribution curve. *p*-Values less than 0.05 were regarded as statistically significant. Bonferroni correction was used for multiple comparisons.

## Figures and Tables

**Table 1:** The clinical, biochemical, and anthropometric parameters of patients depending on the presence of MetS, Me (Q1; Q3).

Indicators	Patients with MetS (*n* = 46)	Patients without MetS (*n* = 64)	*p*-Value
Age, years	39.5 (30; 51)	33 (27.5; 38)	0.001 *
Gender (male, *n* (%)/female, *n* (%))	28 (60.9)/18 (39.1)	30 (46.9)/34 (53.1)	0.340
Duration of disease, years	16 (10; 22)	9 (5; 15)	0.001 *
Glucose, mmol/L	5.20 (4.70; 6.00)	4.90 (4.50; 5.27)	0.003 *
Insulin, μIU/mL	17.99 (14.59; 23.01)	12.98 (9.44; 18.50)	0.008 *
HOMA-IR,	3.96 (2.88; 6.34)	2.73 (1.88; 4.15)	0.004 *
Total cholesterol, mmol/L	4.68 (4.02; 5.45)	4.40 (3.83; 5.11)	0.363
Triglycerides, mmol/L	2.02 (1.74; 2.50)	1.10 (0.80; 1.40)	<0.001 *
High-density lipoproteins, mmol/L	0.90 (0.76; 1.10)	1.20 (0.91; 1.40)	<0.001 *
Low-density lipoproteins, mmol/L	2.67 (1.90; 3.50)	2.70 (2.25; 3.16)	0.572
Atherogenic index	4.17 (3.18; 5.39)	2.82 (2.13; 3.66)	<0.001 *
Waist circumference, cm	106 (100; 114)	86.5 (77; 95)	<0.001 *
Body mass index	31.95 (27.65; 36.05)	23.80 (21.90; 28.00)	<0.001 *
Body fat percentage result	37.3 (30.9; 47.8)	29.9 (21.2; 38.9)	<0.001 *
Visceral fat level	10 (9; 13.5)	6 (4; 8)	<0.001 *
Total fat fold, mm	116.0 (93.5; 140.5)	79.0 (58.5; 97.5)	<0.001 *

Me (Q1; Q3), median (lower quartile; upper quartile); HOMA-IR, homeostasis model assessment of insulin resistance. * *p* < 0.05, statistically significant difference. Comparisons between groups were performed using the chi-square test for gender and the Mann–Whitney *U* test for the other indicators.

**Table 2:** Concentration of adipocytokines in the serum of patients depending on the presence of MetS, Me (Q1; Q3).

Indicators	Patients with MetS (*n* = 46)	Patients without MetS (*n* = 64)	*p*-Value
Leptin, pg/mL	13.51 (7.44; 27.77)	6.66 (2.15; 11.38)	<0.001 *
Adiponectin, μg/mL	6.39 (4.71; 8.97)	11.33 (7.18; 14.65)	0.002 *
Leptin–adiponectin ratio	3.31 (0.76; 5.00)	0.77 (0.37; 2.00)	0.002 *
TNF-α, pg/mL	20.75 (15.38; 26.15)	18.39 (12.22; 24.70)	0.169
IL-6, pg/mL	5.16 (4.00; 8.25)	6.03 (4.60; 7.92)	0.302

Me (Q1;Q3), median (lower quartile; upper quartile); TNF-α, tumor necrosis factor-alpha; IL-6, interleukin 6. * *p* < 0.05, statistically significant difference. Comparisons between groups were performed using the Mann–Whitney *U* test.

**Table 3:** Concentration of adipocytokines in the serum of patients without MetS depending on the number of MetS components, Me (Q1; Q3).

Indicators	0 MetS Components (*n* = 15)	1 MetS Component (*n* = 24)	2 MetS Components (*n* = 25)	*p*-Value
Adiponectin, μg/mL	12.33 (11.53; 21.38)	10.11 (7.14; 13.69)	8.37 (5.96; 14.66)	*p*_01_ = 0.1456 *p*_12_ = 0.7441 *p*_02_ = 0.1113
Leptin, pg/mL	3.14 (1.01; 6.62)	6.87 (2.48; 12.27)	10.89 (3.52; 23.46)	*p*_01_ = 0.0593 *p*_12_ = 0.5003 *p*_02_ = 0.0146 *
Leptin–adiponectin ratio	0.25 (0.06; 0.52)	0.96 (0.47; 2.28)	1.48 (0.43; 3.29)	*p*_01_ = 0.0147 * p_12_ = 0.6905 *p*_02_ = 0.0323
TNF-α, pg/mL	16.09 (10.11; 20.23)	20.12 (13.20; 28.24)	17.98 (13.13; 28.66)	*p*_01_ = 0.0530 *p*_12_ = 0.6938 *p*_02_ = 0.1694
IL-6, pg/mL	4.99 (4.14; 7.26)	5.52 (3.45; 12.23)	5.15 (4.15; 7.24)	*p*_01_ = 0.4529 *p*_12_ = 0.3770 *p*_02_ = 0.9523

Me (Q1;Q3), median (lower quartile; upper quartile); *p*_01_, comparison of patients with 0 and 1 MetS components; *p*_12_, comparison of patients with 1 and 2 MetS components; *p*_02_, comparison of patients with 0 and 2 MetS components. * Statistically significant difference according to the Mann–Whitney *U* test with Bonferroni correction for the three groups of comparisons.

**Table 4:** Concentration of adipocytokines in the serum of patients with MetS depending on the number of MetS components, Me (Q1; Q3).

Indicators	3 MetS Components (*n* = 19)	4 MetS Components (*n* = 22)	*p*-Value
Adiponectin, μg/mL	6.26 (4.71; 8.97)	6.86 (3.37; 8.16)	0.7220
Leptin, pg/mL	10.72 (3.68; 19.58)	19.99 (8.16; 33.18)	0.1396
Leptin–adiponectin ratio	1.20 (0.51; 3.34)	4.03 (3.59; 6.66)	0.0041 *
TNF-α, pg/mL	21.51 (17.83; 27.47)	20.52 (14.66; 25.48)	0.3079
IL-6, pg/mL	5.64 (4.60; 6.23)	7.66 (4.79; 9.91)	0.0552

Me (Q1;Q3), median (lower quartile; upper quartile); * Statistically significant difference according to the Mann–Whitney *U* test with Bonferroni correction for the three groups of comparisons.

## Appendix A

**Table A1:** Spearman’s correlation analysis of the adipocytokines in patients with schizophrenia and metabolic syndrome.

Indicators	Glucose	Insulin	HOMA-IR	High-Density Lipoproteins	Atherogenic Index	Waist Circumference	BMI	The Body Fat Percentage Result	Total Fat Fold
Adiponectin				*r* = 0.620, *p* = 0.001	*r* = −0.526, *p* = 0.003				
Leptin	*r* = 0.445, *p* = 0.002					*r* = 0.298, *p* = 0.044	*r* = 0.442, *p* = 0.003	*r* = 0.669, *p* = 0.001	*r* = 0.606, *p* = 0.001
Leptin–adiponectin ratio		*r* = 0.488, *p* = 0.01	*r* = 0.472, *p* = 0.01			*r* = 0.414, *p* = 0.02	*r* = 0.464, *p* = 0.01	*r* = 0.443, *p* = 0.02	*r* = 0.651, *p* = 0.001
TNF-α		*r* = 0.327, *p* = 0.030	*r* = 0.336, *p* = 0.024						

BMI, body mass index; *r*, Spearman’s correlation coefficient.

**Table A2:** Spearman’s correlation analysis of the adipocytokines in patients with schizophrenia without metabolic syndrome.

Indicators	Insulin	HOMA-IR	Triglycerides	High-Density Lipoproteins	Low-Density Lipoproteins	Atherogenic Index	Waist Circumference	BMI	Body Fat Percentage	Visceral Fat Level	Total Fat Fold
Adiponectin							*r* = −0.343, *p* = 0.041				
Leptin	*r* = 0.405, *p* = 0.006	*r* = 0.416, *p* = 0.004	*r* = −0.462, *p* = 0.001				*r* = 0.527, *p* = 0.001	*r* = 0.712, *p* = 0.001	*r* = 0.820, *p* = 0.001	*r* = 0.502, *p* = 0.001	*r* = 0.696, *p* = 0.001
Leptin–adiponectin ratio	*r* = 0.565, *p* = 0.001	*r* = 0.550, *p* = 0.001	*r* = 0.498, *p* = 0.004			*r* = 0.411, *p* = 0.02	*r* = 0.567, *p* = 0.001	*r* = 0.602, *p* = 0.001	*r* = 0.671, *p* = 0.001		*r* = 0.496, *p* = 0.001
IL-6					*r* = 0.317, *p* = 0.012						
TNF-α				*r* = −0.311, *p* = 0.014		*r* = −0.347, *p* = 0.006					

BMI, body mass index; r, Spearman’s correlation coefficient.
